# Reconstruction of Undersampled Big Dynamic MRI Data Using Non-Convex Low-Rank and Sparsity Constraints

**DOI:** 10.3390/s17030509

**Published:** 2017-03-03

**Authors:** Ryan Wen Liu, Lin Shi, Simon Chun Ho Yu, Naixue Xiong, Defeng Wang

**Affiliations:** 1 Department of Imaging and Interventional Radiology, The Chinese University of Hong Kong, Shatin 999077, N.T., Hong Kong, China; lwsunlight@gmail.com (R.W.L.); simonyu@cuhk.edu.hk (S.C.H.Y.); 2 Department of Medicine and Therapeutics, The Chinese University of Hong Kong, Shatin 999077, N.T., Hong Kong, China; shilin@cuhk.edu.hk; 3 Chow Yuk Ho Technology Center for Innovative Medicine, The Chinese University of Hong Kong, Shatin 999077, N.T., Hong Kong, China; 4 Department of Mathematics and Computer Science, Northeastern State University, Tahlequah, OK 74464, USA; 5 Research Center for Medical Image Computing, The Chinese University of Hong Kong, Shatin 999077, N.T., Hong Kong, China; 6 Shenzhen Research Institute, The Chinese University of Hong Kong, Shenzhen 518057, China

**Keywords:** compressed sensing, dynamic magnetic resonance imaging, low-rank, non-convex optimization, robust principal component analysis

## Abstract

Dynamic magnetic resonance imaging (MRI) has been extensively utilized for enhancing medical living environment visualization, however, in clinical practice it often suffers from long data acquisition times. Dynamic imaging essentially reconstructs the visual image from raw (**k**,*t*)-space measurements, commonly referred to as big data. The purpose of this work is to accelerate big medical data acquisition in dynamic MRI by developing a non-convex minimization framework. In particular, to overcome the inherent speed limitation, both non-convex low-rank and sparsity constraints were combined to accelerate the dynamic imaging. However, the non-convex constraints make the dynamic reconstruction problem difficult to directly solve through the commonly-used numerical methods. To guarantee solution efficiency and stability, a numerical algorithm based on Alternating Direction Method of Multipliers (ADMM) is proposed to solve the resulting non-convex optimization problem. ADMM decomposes the original complex optimization problem into several simple sub-problems. Each sub-problem has a closed-form solution or could be efficiently solved using existing numerical methods. It has been proven that the quality of images reconstructed from fewer measurements can be significantly improved using non-convex minimization. Numerous experiments have been conducted on two in vivo cardiac datasets to compare the proposed method with several state-of-the-art imaging methods. Experimental results illustrated that the proposed method could guarantee the superior imaging performance in terms of quantitative and visual image quality assessments.

## 1. Introduction

Magnetic resonance imaging (MRI) has been established as an advanced non-invasive diagnostic imaging technique for visualizing the structure and functionality of the human body [1]. Many efforts have been made to dramatically improve the imaging speed and quality. In particular, the imaging of dynamic medical information plays an important role in enhancing medical living environment visualization [2,3,4,5,6,7]. Dynamic imaging has been becoming more and more important for several clinical applications, such as perfusion [8], cardiac [9] and functional imaging [10]. A large number of advanced image processing techniques (e.g., segmentation [11,12,13], classification [14,15,16], denoising [17,18] and deconvolution [19,20], etc.) have been developed based on MRI data to assist medical diagnosis. 

MRI often suffers from long data acquisition times in clinical practice. Essentially, dynamic MRI, which reconstructs the visual image from raw (**k**,*t*)-space measurements, is commonly referred to as big data computing. The inherently slow acquisition speed often makes it difficult for dynamic MRI to capture high spatio-temporal resolutions, high signal-to-noise ratio, and proper volume coverage. The low spatio-temporal resolutions easily lead to erroneous diagnostic information and inaccurate estimation of tracer kinetic parameters, especially for dynamic susceptibility contrast MRI (DSC-MRI) [21]. The current undersampled reconstruction methods are of importance for the acceleration of imaging times in dynamic MRI. The spatio-temporal resolutions could be improved accordingly to capture the rapid tracer kinetics and enhance the diagnostic accuracy [22]. In particular, the accurate estimation of tracer kinetics plays a crucial role in quantifying the cerebral blood flow (CBF), cerebral blood volume (CBV) and mean transit time (MTT) to assess brain perfusion [23]. The high spatio-temporal resolutions could provide more medical information which is able to assist physicians in making an accurate diagnosis. Thus, there is a great potential to develop advanced imaging techniques to accelerate dynamic MRI in clinical practice.

Compressed sensing (CS) [24,25,26,27,28,29], which exploits the fact that an image can be sparsely represented in a certain transform domain, has been successfully used in dynamic MRI, such as **k**-t SPARSE [30], **k**-*t* FOCUSS [31,32] and **k**-t SPARSE-SENSE [33]. Note that the dynamic MRI sequence is both spatially and temporally correlated. The reconstructed results yielded by above CS-based methods may suffer from low spatial or temporal resolution. The purpose of this study is to accelerate dynamic MRI with high spatial and temporal resolutions. Recently, many efforts have been made to exploit the low-rank property of dynamic MRI sequence, instead of its only sparsity. Inherently, low-rank matrix completion can be considered as an effective extension of CS, which recoveries the missing or corrupted entries of a matrix under low-rank and incoherent conditions [34,35,36,37]. For instance, Lingala et al. [8,38] and Zhao et al. [39] have investigated the low-rank and sparse properties of the Casorati matrix (i.e., spatio-temporal MRI signal) and combined them together to further accelerate the dynamic MRI. The low-rank plus sparse decomposition model, referred to as robust principal component analysis (RPCA) [40,41], has also attracted increasing attention recently. Many results in the literature showed that it is possible to recover both low-rank and sparse components from a few incoherent observations under some assumptions [40,42,43].

Motivated by the theory of RPCA, Trémoulhéac et al. [44] proposed to reconstruct the dynamic MRI as the sum of low-rank plus sparse components. In this case, the dynamic MRI reconstruction problem was formulated as a least-squares optimization problem which was regularized by convex low-rank and sparsity constraints. Experimental results have demonstrated the effectiveness under different imaging conditions. The combination of low-rank with sparsity constraints can significantly improve the dynamic imaging result compared with the reconstruction models regularized by low-rank or sparsity constraint alone. It is generally thought that the superior performance of the combination version benefits from the full use of MRI data coherence and redundancy. Current studies [38,45,46,47] have shown that non-convex minimization could generate sparser solutions and provide better reconstruction in practice. It inherently means that the potential solutions can be sparsely represented in certain transform domains. Non-convex minimization has gained increasing attention in the fields of numerical optimization, image processing and computer version [48,49,50,51]. To make non-convex minimization more practical, a large number of numerical methods have been presented to solve the corresponding optimization problem. Inspired by success of non-convex optimization, there is a great potential to use the non-convex Schatten-*p* norm (0<p<1) as the low-rank constraint. In addition, to further enhance the sparse component recovery, non-convex *L_q_* quasi-norm (0<q<1) can be effectively adopted to yield better result compared with commonly-used convex *L*_1_-norm [52,53,54,55,56]. In this paper, non-convex low-rank and sparsity constraints will be incorporated into our dynamic MRI reconstruction framework to improve imaging speed and quality.

In this work, we propose to formulate the undersampled dynamic MRI reconstruction as a least-squares optimization problem regularized by non-convex low-rank and sparsity constraints. The quality of the reconstructed images can be significantly improved by taking advantages of these constraints. However, due to the non-convex and non-smooth natures of the constraints, it is difficult to directly solve the resulting optimization problem through commonly-used numerical methods. To guarantee solution stability and efficiency, a numerical optimization algorithm based on Alternating Direction Method of Multipliers (ADMM) is proposed to solve the resulting optimization problem. In particular, the original non-convex non-smooth optimization problem will be decomposed into several subproblems by introducing several intermediate variables. These subproblems have simple closed-form solutions or could be efficiently handled using existing numerical methods. Thus, the main contributions of this paper mainly rely on the non-convex dynamic MRI reconstruction model and its numerical optimization algorithm. Experimental results on two in vivo cardiac MRI datasets have verified the effectiveness of the proposed method in terms of both quantitative and qualitative image quality evaluations.

The remainder of this paper is organized into several sections. The following section briefly introduces the basic concepts of dynamic MRI and robust principal component analysis. In Section 3, we tend to develop a non-convex minimization framework for accelerating dynamic MRI data acquisition using both non-convex low-rank and sparsity constraints. The resulting optimization algorithm is efficiently solved using an ADMM-based numerical algorithm. Numerous experiments on two in vivo cardiac datasets are performed in Section 4. Finally, we conclude this work in Section 5 by summarizing our contributions.

## 2. Background

### 2.1. Dynamic MRI from Partial Measurements

We denote the dynamic MRI to be reconstructed as a spatio-temporal signal I(x,t), where x is the spatial coordinate and t denotes time. The imaging equation in dynamic MRI is defined as follows:

(1) $$S\left(k,t_{i}\right)=\int^{\text{​}}I\left(x,t_{i}\right)\exp\left(-j2\pi \left(k\cdot x\right)\right)dx+N\left(k,t_{i}\right)$$

for i=1,2,⋯,T, where S(k,t) denotes the measured (k,t)-space signal, and N(k,t) is assumed to be additive complex-valued Gaussian noise. For the sake of simplicity, we consider a discrete image model in this dissertation. Given $N_{t}$ MR images of dimension $N_{x}\times N_{y}$. The spatio-temporal signal I(x,t) can be rearranged into a matrix form, i.e.,

$$F=\left[\begin{array}{ccc} I\left(1,t_{1}\right) & \cdots & I\left(1,t_{N_{t}}\right)\\ \vdots & \ddots & \vdots \\ I\left(N_{x}N_{y},t_{1}\right) & \cdots & I\left(N_{x}N_{y},t_{N_{t}}\right) \end{array}\right]\in {\mathbb{C}}^{N_{x}N_{y}\times N_{t}}$$

The Casorati matrix F, whose first and second directions respectively represent the spatial and temporal dimensions, is approximately low-rank due to the strong correlation existed between dynamic MR images [38]. Thus, a finite-dimensional spatio-temporal MRI model equivalent to Equation (1) can be written as:

(2) d=𝓐(F)+n

where $d\in {\mathbb{C}}^{P}$ denotes the vector of the stacked (k,t)-space measurements, $𝓐:\;{\mathbb{C}}^{N_{x}N_{y}\times N_{t}}\to {\mathbb{C}}^{P}$ is the undersampled Fourier transform operator with $P\ll N_{x}N_{y}\times N_{t}$, $n\in {\mathbb{C}}^{P}$ is the Gaussian noise vector. Recovering the matrix F from a limited number of measurements d is a typical ill-conditional problem. To cope with the ill-conditional nature, there has been considerable interest in exploiting low-rank and sparisty of the dynamic images to enhance the reconstruction accuracy [35]. Thus, there is a great potential to combine low-rank with sparsity constraints to improve dynamic MRI from partial measurements.

### 2.2. Robust Principal Component Analysis

Recent work has shown that robust principal component analysis (RPCA) [40,41] is capable of decomposing a Casorati matrix X into a low-rank component **L** and a sparse component **S** by constraining the rank of **L** and the sparsity of S simultaneously. It is worth noting that RPCA can be regarded as a robust extended version of traditional PCA [57]. PCA is performed based on the basic assumption of additive Gaussian noise and uses the sum of squared differences as the loss function. Theoretically, it works well as long as the value of noise is small enough. However, if the data are perturbed by high-level noise, it will be impossible to generate satisfactory reconstruction results since the traditional PCA could be easily corrupted by these gross errors [58]. It is well known that raw (**k**,*t*)-space data are often obtained from the MRI machines under complex imaging conditions. The raw data may be significantly corrupted by the outliers and large noise in clinical practice. If we directly adopt PCA to reconstruct the MRI images, it will be difficult to guarantee high-quality reconstruction due to the inherent limitation of PCA. In contrast, RPCA can perform well when the observed data are corrupted by severe perturbations because of its robust properties. The recent theoretical results indicate that the low-rank matrix can be exactly recovered by considering the following constrained convex minimization problem:

(3) $$\underset{L,S}{\min}\;{\left\|L\right\|}_{*}+\rho {\left\|S\right\|}_{1}\;\mathrm{such}\;\mathrm{that}\;X=L+S$$

where ρ is a weight parameter, the nuclear norm ${\left\|L\right\|}_{*}$ is defined as ${\left\|L\right\|}_{*}=\sum_{i=1}^{r}\sigma_{i}$ with $\sigma_{1},\;\sigma_{2},\cdots ,\sigma_{r}$ being the singular values of **L** and *r* being the rank of **L**. In the literature, the classical ADMM [59] can be exploited to efficiently solve the RPCA Problem (3) based on the following augmented Lagrangian function:

(4) $${𝓛}_{A}=\;{\left\|L\right\|}_{*}+\rho {\left\|S\right\|}_{1}+\frac{\alpha }{2}\|X-\left(L+S\right)+\frac{Z}{\alpha }{\|}_{2}^{2}$$

where Z denotes the Lagrange multiplier and α is a positive penalty parameter. ADMM makes full use of the separable structure of Equation (4), and decomposes Equation (4) over primal variables **L** and **S**. The iterative procedure is detailedly summarized in Algorithm 1. 

**Algorithm 1.** RPCA1 **Input**: Casorati matrix
X, decomposition parameter ρ>02 **Initialize**:
$S^{0}=Z^{0}=0$, *k*=03 **while**
*stopping criterion is not satisfied*
**do**4  $L^{k+1}=T_{\alpha^{-1}}\left(X-S^{k}+\alpha^{-1}Z^{k}\right)$5  $S^{k+1}=S_{\rho /\alpha }\left(X-L^{k+1}+\alpha^{-1}Z^{k}\right)$6  $Z^{k+1}=Z^{k}+\alpha \left(X-L^{k+1}-S^{k+1}\right)$7 **end while**8 **Output**:
$\left(L^{*},S^{*}\right)$

In particular, ADMM minimizes ${𝓛}_{A}$ in Equation (4) over **L** and **S** separately, and then updates the Lagrange multiplier Z. The corresponding **L**-subproblem and **S**-subproblem are given by:

(5) $$L^{k+1}=\underset{L}{\min\;}{\left\|L\right\|}_{*}+\frac{\alpha }{2}\|\left(X-S^{k}+\frac{Z^{k}}{\alpha }\right)-L{\|}_{2}^{2}$$

(6) $$S^{k+1}=\underset{S}{\min\;}\rho {\left\|S\right\|}_{1}+\frac{\alpha }{2}\|\left(X-L^{k+1}+\frac{Z^{k}}{\alpha }\right)-S{\|}_{2}^{2}$$

where k represents the iteration number. The closed-form solutions to Equations (5) and (6) are respectively obtained using the singular value thresholding operator $T_{\tau }\left(Y\right)=US_{\tau }\left(\Xi \right)V^{H}$ (where $Y=U\Xi V^{H}$ denotes the singular value decomposition) and shrinkage operator $S_{\tau }\left(Y\right)=sign\left(Y\right)\max\left(\left|Y\right|-\tau ,0\right)$, i.e.,

(7) $$L^{k+1}=T_{\alpha^{-1}}\left(X-S^{k}+\alpha^{-1}Z^{k}\right)$$

(8) $$S^{k+1}=S_{\rho /\alpha }\left(X-L^{k+1}+\alpha^{-1}Z^{k}\right)$$

In Equations (7) and (8), the definition of sign function sign(·) is given by:

$$sign\left(s\right)=\{\begin{array}{r} 1,\;s>0\\ 0,\;s=0\\ -1,\;s<0 \end{array}$$

Given the fixed values of $L^{k+1}$ and $S^{k+1}$, the Lagrange multiplier Z is accordingly updated as $Z^{k+1}=Z^{k}+\alpha \left(X-L^{k+1}-S^{k+1}\right)$**.** The parameter ρ can be seen as a trade-off between low-rank and sparse components. The theoretically suggested value introduced in [40] is set to $\rho =1/\sqrt{\max\left(N_{x}N_{y},N_{t}\right)}$, where $N_{x}N_{y}$ and $N_{t}$ denote the number of pixels in each frame and the number of time-frames in Casorati matrix X, respectively. 

## 3. k-*t* NCRPCA: Formulation

### 3.1. Joint Non-Convex Low-Rank and Sparsity Constraints

**Figure 1:**
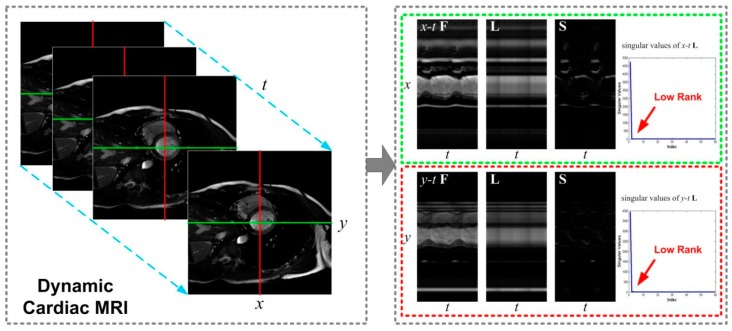
The low-rank plus sparse (**L** + **S**) decomposition of a fully sampled 2D axial cardiac MRI dataset. RPCA decomposes the x-t (or *y*-*t*) temporal profiles (i.e., Casorati matrix) **F** into a low-rank component **L** and a sparse component **S**. The singular values of both *x*-*t* and *y*-*t* **L** tend to zero quickly as their index increases. **S** can be effectively represented using a sparsifying transform.

In this study, it is assumed that the Casorati matrix **F** can be decomposed as a low-rank component L and a sparse component S. Figure 1 shows the L+S decomposition of an axial cardiac MRI dataset, where L captures the corrected background between time frames and S captures the temporal or dynamic information. 

In can be observed that the singular values of **L** tend to zero quickly as their index increases, and **S** can be effectively represented using a sparsifying transform. Based on the spatio-temporal MRI Model (2) and RPCA (3), Trémoulhéac et al. [44] proposed to reconstruct the dynamic MRI F as the sum of low-rank L plus sparse S components. The dynamic MRI reconstruction can be formulated as a least squares optimization problem regularized by convex low-rank and sparsity constraints, i.e.,

(9) $$\underset{L,S}{\min}\frac{1}{2}\|𝓐\left(L+S\right)-d{\|}_{2}^{2}+\mu_{1}{\left\|L\right\|}_{*}+\mu_{2}\|F_{t}\left(S\right){\|}_{1}$$

where both $\mu_{1}$ and $\mu_{2}$ are positive regularization parameters, and $F_{t}$ represents the Fourier transform operator along the temporal direction. The assumption behind the use of this sparsifying transform is that the dynamic MRI in time exhibits strong correlation or periodicity. The unclear norm ${\left\|\circ \right\|}_{*}$ is equivalent to the *L*_1_-norm ${\left\|\circ \right\|}_{1}$ because the singular values are all nonnegative. Current studies in the literature [38,45,46,47] illustrated that non-convex minimization could yield sparser solutions and provide consistently better performance over convex minimization. Thus, there is a great potential to use non-convex Schatten-*p* norm (0<p<1) as the low-rank constraint. To improve the sparse component recovery, non-convex *L_q_* quasi-norm (0<q<1) can yield better recovery results over commonly-used convex *L*_1_-norm [52,53,54,55]. Motivated by the success of non-convex *L_q_* quasi-norm, we propose to replace the convex *L*_1_-norm $\|F_{t}{\left(S\right)\|}_{1}$ by its non-convex version $\|F_{t}{\left(S\right)\|}_{q}^{q}$ in Equation (9) to further improve the quality of reconstructed image.

With the above notations, we propose to formulate undersampled dynamic MRI reconstruction as a least-squares optimization problem regularized by non-convex low-rank and sparsity constraints, i.e.,

(10) $$\underset{L,S}{\min}\frac{1}{2}\|𝓐\left(L+S\right)-d{\|}_{2}^{2}+\mu_{1}{\left\|L\right\|}_{p}^{p}+\mu_{2}\|F_{t}\left(S\right){\|}_{q}^{q}$$

where ${\left\|L\right\|}_{p}^{p}=\sum_{i=1}^{\min\left\{N_{x}N_{y},N_{t}\right\}}\sigma_{i}^{p}\left(L\right)$ for some p∈(0,1) denotes the non-convex low-rank constraint in which $\sigma_{i}\left(L\right)$ is the *i*th largest singular value of L and $\min\left\{N_{x}N_{y},N_{t}\right\}$ is the rank of the matrix L. The non-convex sparsity constraint, defined as $\|F_{t}\left(S\right){\|}_{q}^{q}=\sum_{i=1}^{N_{x}N_{y}N_{t}}{\left|{\left(F_{t}\left(S\right)\right)}_{i}\right|}^{q}$ for some q∈(0,1), is used to promote sparsity in Fourier transform domain. The positive regularization parameters $\mu_{1}$ and $\mu_{2}$ are adjusted to balance the trade-off between the data-fidelity term and the joint non-convex low-rank and sparsity constraints. The sparsifying transform $F_{t}$ is incoherent with the Fourier sampling operator 𝓐, thus the proposed regularized reconstruction is well posed in practice.

### 3.2. Numerical Optimization Algorithm

The resulting optimization Problem (10) includes both non-convex low-rank and sparsity constraints. The introduced non-convex constraints make dynamic reconstruction problem difficult to solve. Thus, it is intractable to solve Problem (10) directly using current minimization schemes. To guarantee solution stability and efficiency, an ADMM-based optimization algorithm is proposed in this work. Two auxiliary variables P=L and $Q=F_{t}\left(S\right)$ are first introduced and the unconstrained minimization Problem (10) is then transformed into the following constrained version:

(11) $$\underset{P,Q,L,S}{\min}\frac{1}{2}\|𝓐\left(L+S\right)-d{\|}_{2}^{2}+\mu_{1}\|P{\|}_{p}^{p}+\mu_{2}\|Q{\|}_{q}^{q}\;\mathrm{subject}\;\mathrm{to}\;P=L,\;Q=F_{t}\left(S\right)$$

whose associated augmented Lagrangian function is given by:

(12) $${𝓛}_{A}=\frac{1}{2}\|𝓐\left(L+S\right)-d{\|}_{2}^{2}+\mu_{1}\|P{\|}_{p}^{p}+\mu_{2}\|Q{\|}_{q}^{q}+\frac{\alpha_{1}}{2}\|L-P+\frac{Z_{1}}{\alpha_{1}}{\|}_{2}^{2}+\frac{\alpha_{2}}{2}\|F_{t}\left(S\right)-Q+\frac{Z_{2}}{\alpha_{2}}{\|}_{2}^{2}$$

where $Z_{1}$ and $Z_{2}$ denote the Lagrange multipliers, $\alpha_{1}$ and $\alpha_{2}$ represent positive penalty parameters that control the weights of penalty terms. As $\alpha_{1},\alpha_{2}\to \infty$, solution of the above Problem (12) tends to that of Problem (11).

Since the variables P, Q, L and S are coupled together in the augmented Lagrangian Function (12), it is computationally intractable to solve them simultaneously. ADMM minimizes ${𝓛}_{A}$ over P, Q, L and S separately leading to several subproblems which have closed-form solutions or could be efficiently solved using existing numerical methods. This decouples the individual updates of P, Q, L and S, therefore the original optimization task can be simplified as follows:

(13) $$P^{k+1}=\underset{P}{\min\;}\mu_{1}\|P{\|}_{p}^{p}+\frac{\alpha_{1}}{2}\|P-\left(L^{k}+Z_{1}^{k}/\alpha_{1}\right){\|}_{2}^{2}$$

(14) $$Q^{k+1}=\underset{Q}{\min\;}\mu_{2}\|Q{\|}_{q}^{q}+\frac{\alpha_{2}}{2}\|Q-\left(F_{t}\left(S^{k}\right)+Z_{2}^{k}/\alpha_{2}\right){\|}_{2}^{2}$$

(15) $$L^{k+1}=\frac{1}{2}\|𝓐\left(L+S^{k}\right)-d{\|}_{2}^{2}+\frac{\alpha_{1}}{2}\|L-\left(P^{k+1}-Z_{1}^{k}/\alpha_{1}\right){\|}_{2}^{2}$$

(16) $$S^{k+1}=\frac{1}{2}\|𝓐\left(L^{k+1}+S\right)-d{\|}_{2}^{2}+\frac{\alpha_{2}}{2}\|F_{t}\left(S\right)-\left(Q^{k+1}-Z_{2}^{k}/\alpha_{2}\right){\|}_{2}^{2}$$

The first P-subproblem (13) can be considered as a similar form of the standard unclear norm minimization problem. To achieve an efficient solution, the iterative singular value thresholding scheme [60,61], originally proposed for unclear norm minimization, can be extended to solve (13) which has a non-convex low-rank constraint. Let ${\tilde{P}}^{k}=L^{k}+Z_{1}^{k}/\alpha_{1}$, the solution $P^{k+1}$ is given by:

(17) $$P^{k+1}=\sum_{i=1}^{\min\left\{N_{x}N_{y},N_{t}\right\}}\max\left(\sigma_{i}-\frac{\mu_{1}\sigma_{i}^{p-1}}{\alpha_{1}},0\right)u_{i}v_{i}^{T}$$

where the superscript T denotes the transpose (conjugate transpose) operator for real (complex) matrices or vectors. In Equation (17), $u_{i}$, $v_{i}$ and $\sigma_{i}$ respectively represent the singular vectors and values of ${\tilde{P}}^{k}$. Note that, if p=1, the expression in Equation (17) can be regarded as equivalent to a standard shrinkage algorithm for nuclear norm optimization problem.

It is difficult to directly solve the second subproblem (14) owing to the non-convex sparsity constraint. Motivated by the successful applications of soft thresholding [62] and iterative shrinkage/thresholding [63], a generalized iterated shrinkage algorithm (GISA) algorithm [64] is introduced in this study to efficiently solve the non-convex minimization problem. In particular, the Q-subproblem (14) can be decoupled into $N_{x}N_{y}\times N_{t}$ independent and unconstrained subproblems. The standard version of these subproblems is given by:

(18) $$y^{*}=\underset{y}{\min\;}\tilde{\mu }{\left|y\right|}^{q}+\frac{1}{2}{\left(y-c\right)}^{2}$$

where $\tilde{\mu }$ is a positive parameter, y denotes the scalar variable that needs to be estimated, and c is a known scalar constant. As discussed in [64], the proposed GISA algorithm can be adopted to efficiently solve the non-convex Problem (18) with high accuracy. Therefore, the optimal solution of (18) can be obtained as follows:

(19) $$y^{*}=\{\begin{array}{cc} 0, & \;\mathrm{if}\;\left|c\right|\leq \tau_{\tilde{\mu }}\\ \mathrm{sign}\left(c\right)ST_{q}\left(\left|c\right|,\;\tilde{\mu }\right), & \;\mathrm{if}\;\left|c\right|>\tau_{\tilde{\mu }} \end{array}$$

where:

$$\tau_{\tilde{\mu }}={\left(2\tilde{\mu }\left(1-q\right)\right)}^{1/\left(2-q\right)}+\tilde{\mu }q{\left(2\tilde{\mu }\left(1-q\right)\right)}^{\left(q-1\right)/\left(2-q\right)}$$

and $ST_{q}\left(\left|c\right|,\;\tilde{\mu }\right)$ can be obtained by iteratively performing the following equation:

$$ST_{q}\left(\left|c\right|,\;\tilde{\mu }\right)-\left|c\right|+\tilde{\mu }q{\left(ST_{q}\left(\left|c\right|,\;\tilde{\mu }\right)\right)}^{q-1}=0$$

Thus, solution $Q^{k+1}\;$of the subproblem (14) is given by:

(20) $$Q^{k+1}=\{\begin{array}{cc} 0, & \;\mathrm{if}\;\left|F_{t}\left(S^{k}\right)+Z_{2}^{k}/\alpha_{2}\right|\leq \tau_{\mu_{2}/\alpha_{2}}\\ \mathrm{sign}\left(F_{t}\left(S^{k}\right)+Z_{2}^{k}/\alpha_{2}\right)ST_{q}\left(\left|F_{t}\left(S^{k}\right)+Z_{2}^{k}/\alpha_{2}\right|,\;\frac{\mu_{2}}{\alpha_{2}}\right), & \;\mathrm{if}\;\left|F_{t}\left(S^{k}\right)+Z_{2}^{k}/\alpha_{2}\right|>\tau_{\mu_{2}/\alpha_{2}} \end{array}$$

Both the third and fourth subproblems (15) and (16) are essentially quadratic and thus can be solved analytically as:

(21) $$L^{k+1}={\left({𝓐}^{⊺}𝓐+\alpha_{1}I\right)}^{-1}\left({𝓐}^{⊺}d+\alpha_{1}P^{k+1}-Z_{1}^{k}-{𝓐}^{⊺}𝓐S^{k}\right)$$

(22) $$S^{k+1}={\left({𝓐}^{⊺}𝓐+\alpha_{2}I\right)}^{-1}\left({𝓐}^{⊺}d+F_{t}^{⊺}\left(\alpha_{2}Q^{k+1}-Z_{2}^{k}\right)-{𝓐}^{⊺}𝓐L^{k+1}\right)$$

For fixed values of $P^{k+1}$, $Q^{k+1}$, $L^{k+1}$ and $S^{k+1}$, the Lagrange multipliers $Z_{1}$ and $Z_{2}$ are updated as follows:

(23) $$Z_{1}^{k+1}=Z_{1}^{k}-\alpha_{1}\left(P^{k+1}-\;L^{k+1}\right)$$

(24) $$Z_{2}^{k+1}=Z_{2}^{k}-\alpha_{2}\left(Q^{k+1}-\;F_{t}\left(S^{k+1}\right)\right)$$

Based on these analytic solutions, the proposed image reconstruction procedure is detailedly summarized in Algorithm 2. The original non-convex optimization problem is decomposed into four subproblems. Each of these subproblems has a closed-form solution or could be efficiently solved using existing numerical method. Due to the non-convex and non-smooth constraints, it is difficult to yield the resulting theoretical convergence result. However, the quality of reconstructed image could be improved using the joint non-convex low-rank and sparsity constraints. In this study, the proposed method is referred to as **k**-*t* NCRPCA for dynamic MRI reconstruction from undersampled (k,t)-space measurements.

**Algorithm 2.**
**k**-*t* NCRPCA1 **Input**: Fourier transform 𝓐, (k,t)-space data d and parameters $\left(\mu_{1},\mu_{2},\alpha_{1},\alpha_{2},p,q,\tau_{\alpha_{2},\mu_{2}}\right)$.2 **Initialize**: $F^{0}=L^{0}={𝓐}^{⊺}d$, $S^{0}=Z_{1}^{0}=Z_{2}^{0}=0$.3 **while**
*stopping criterion is not satisfied*
**do**4  $P^{k+1}=\overset{\min\left\{N_{x}N_{y},N_{t}\right\}}{\underset{i=1}{\sum }}\max\left(\sigma_{i}-\frac{\mu_{1}\sigma_{i}^{p-1}}{\alpha_{1}},0\right)u_{i}v_{i}^{⊺}$5  $Q^{k+1}=\{\begin{array}{cc} 0, & \;\mathrm{if}\;\left|F_{t}\left(S^{k}\right)+Z_{2}^{k}/\alpha_{2}\right|\leq \tau_{\mu_{2}/\alpha_{2}}\\ \mathrm{sign}\left(F_{t}\left(S^{k}\right)+Z_{2}^{k}/\alpha_{2}\right)ST_{q}\left(\left|F_{t}\left(S^{k}\right)+Z_{2}^{k}/\alpha_{2}\right|,\frac{\mu_{2}}{\alpha_{2}}\;\right), & \;\mathrm{if}\;\left|F_{t}\left(S^{k}\right)+Z_{2}^{k}/\alpha_{2}\right|>\tau_{\mu_{2}/\alpha_{2}} \end{array}$6  $L^{k+1}={\left({𝓐}^{⊺}𝓐+\alpha_{1}I\right)}^{-1}\left({𝓐}^{⊺}d+\alpha_{1}P^{k+1}-Z_{1}^{k}-{𝓐}^{⊺}𝓐S^{k}\right)$7  $S^{k+1}={\left({𝓐}^{⊺}𝓐+\alpha_{2}I\right)}^{-1}\left({𝓐}^{⊺}d+F_{t}^{⊺}\left(\alpha_{2}Q^{k+1}-Z_{2}^{k}\right)-{𝓐}^{⊺}𝓐L^{k+1}\right)$8  $Z_{1}^{k+1}=Z_{1}^{k}-\alpha_{1}\left(P^{k+1}-\;L^{k+1}\right)$9  $Z_{2}^{k+1}=Z_{2}^{k}-\alpha_{2}\left(Q^{k+1}-\;F_{t}\left(S^{k+1}\right)\right)$10  $F^{k+1}=L^{k+1}+S^{k+1}$11  $\alpha_{1}=1.2\alpha_{1}$ and $\alpha_{2}=1.2\alpha_{2}$12 **end while**13 **Output**:
$\left(F^{*},L^{*},S^{*}\right)$

## 4. Experimental Results and Discussion

Experimental results on in vivo axial and coronal cardiac MRI datasets were conducted in this study to demonstrate the superior performance of our proposed method in terms of both quantitative and visual quality evaluations.

### 4.1. Acquired Datasets

**Figure 2:**
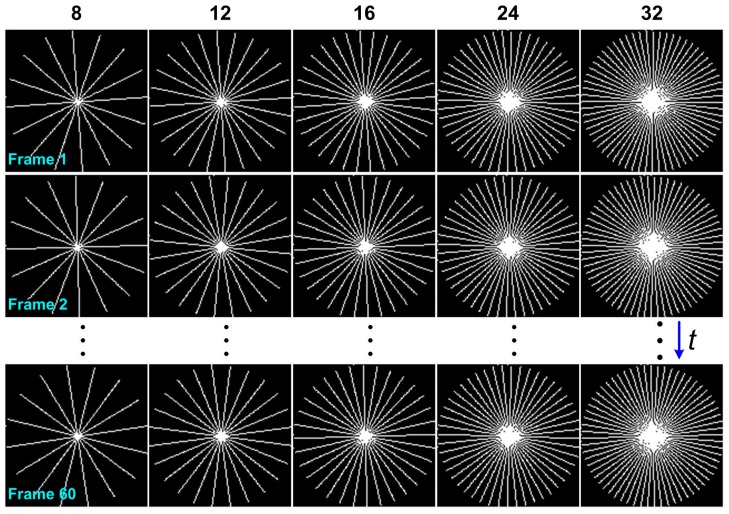
Radial sampling trajectories with N number of uniformly spaced rays in each frame with subsequent random rotations across time frames. From left to right: different number of radial rays ranging from N=8, 12, 16, 24 and 32 was considered to simulate the undersampled (k,t)-space measurements.

To evaluate the performance of dynamic MRI reconstruction, experiments were conducted on both in vivo axial and coronal cardiac datasets. The 2D cardiac cine imaging was performed in a healthy adult volunteer, with the approval from The Joint Chinese University of Hong Kong—New Territories East Cluster Clinical Research Ethics Committee (The Joint CUHK-NTEC CREC). The datasets were acquired from a clinical 3T MRI scanner (Achieva, Philips Medical Systems, Best, The Netherlands) with an eight-channel receiver coil. The relevant imaging parameters were as follows: TR/TE = 3.8/1.9 ms, flip angle = 45°, image matrix size = 128×128, and temporal frames = 60. During cardiac imaging, the volunteer was instructed to hold the breath for as long as possible. As shown in Figure 2, the undersampled (k,t)-space measurements were obtained from 8, 12, 16, 24 and 32 radial projections. To obtain a full Nyquist-sampled dataset, $\frac{\pi }{2}n$ projections (201 for n=128 in our case) should be acquired theoretically. Therefore, the above radial projections respectively correspond to undersampling factors of ~25, 16, 12, 8 and 6. In addition, the radial sampling pattern shown in Figure 2 has uniformly spaced radial rays per frame and subsequent random rotations across time frames for maintaining sampling incoherence. All experiments were implemented in MATLAB^®^ (The MathWorks Inc., Natick, MA, USA) using a computer with 3.1 GHz Intel Core i5-2500 PU, 4 GB RAM. For comparison, the high-quality results reconstructed from fully sampled (k,t)-space measurements were used as ground truth reference in our numerical experiments. The proposed method will be compared with three state-of-the-art reconstruction methods, i.e., **k**-*t* FOCUSS [31,32], **k**-*t* SLR [38] and **k**-*t* RPCA [44]. **k**-*t* FOCUSS is able to take full advantage of the sparse properties of dynamic MRI to enhance reconstruction quality. The last two reconstruction methods were proposed by simultaneously using the low-rank and sparsity constraints. The resulting optimization methods were effectively solved through the alternating direction methods. Note that **k**-*t* RPCA can be considered as a special case of our proposed method (10). The satisfactory reconstructed results were generated by these three methods with the corresponding parameters manually optimized.

### 4.2. Parameters Settings

The accuracy of reconstruction results will be evaluated using the signal to error ratio (SER) [38] and the structural similarity index (SSIM). SSIM is more consistent with the human visual perception. The SER in mathematical form is defined as follows:

(25) $$\mathrm{SER}=\;-10\log_{10}\frac{\|F_{\mathrm{rec}}-F_{\mathrm{truth}}{\|}_{2}^{2}}{\|F_{\mathrm{truth}}{\|}_{2}^{2}}\;\left(\mathrm{dB}\right)$$

where $F_{\mathrm{rec}}$ and $F_{\mathrm{truth}}$ denote the recovered matrix and the ground truth fully-sampled noiseless matrix, respectively. Let $f_{\mathrm{rec}}$ and $f_{\mathrm{truth}}$ represent the time-frame magnitude images extracted from $F_{\mathrm{rec}}$ and $F_{\mathrm{truth}}$, respectively. The definition of SSIM between $f_{\mathrm{rec}}$ and $f_{\mathrm{truth}}$ is given by:

(26) $$\mathrm{SSIM}=\frac{\left(2\mu_{f_{\mathrm{rec}}}\mu_{f_{\mathrm{truth}}}+c_{1}\right)\left(2\sigma_{f_{\mathrm{rec}}f_{\mathrm{truth}}}+c_{2}\right)}{\left(\mu_{f_{\mathrm{rec}}}^{2}+\mu_{f_{\mathrm{truth}}}^{2}+c_{1}\right)\left(\sigma_{f_{\mathrm{rec}}}^{2}+\sigma_{f_{\mathrm{truth}}}^{2}+c_{2}\right)}$$

where $\mu_{f_{\mathrm{rec}}}$ and $\mu_{f_{\mathrm{truth}}}$ represent the local mean values, $\sigma_{f_{\mathrm{rec}}}$ and $\sigma_{f_{\mathrm{truth}}}$ denote the standard deviations, $\sigma_{f_{\mathrm{rec}}f_{\mathrm{truth}}}$ is the covariance value, and $c_{1}$ and $c_{2}$ are two predefined constants to avoid instability with weak denominator. Both SER and SSIM are able to provide quantitative indexes of reconstruction results, but they cannot be well correlated with perceptual quality. Thus, the specific reconstructed frames and the temporal profiles will also be shown to enhance visual comparisons.

The proposed method in Algorithm 2 mainly involves six parameters, i.e., $\mu_{1}$, $\mu_{2}$, $\alpha_{1}$, $\alpha_{2}$, p and q. Inspired by previous work [40], a suggested $\mu_{2}$ is set to $\mu_{2}=\rho \mu_{1}$ with $\rho =1/\sqrt{\max\left(N_{x}N_{y},N_{t}\right)}$, where $N_{x}N_{y}$ and $N_{t}$ respectively denote the number of pixels in each frame and the number of time-frames in Casorati matrix F. The regularization parameter $\mu_{1}$ is manually set as $2\times 10^{2}$ for **k**-*t* NCRPCA. The convergence rate of **k**-*t* NCRPCA is highly dependent on the selection of $\alpha_{1}$ and $\alpha_{2}$. It has been proven that lower values of $\alpha_{1}$ and $\alpha_{2}$ result in a much faster convergence rate. However, the proposed method would suffer from image quality degradation since constraints in Equation (4) are not satisfied. Higher values of $\alpha_{1}$ and $\alpha_{2}$ can guarantee that all constraints can be satisfied, but generally lead to slower convergence. To balance the trade-off between convergence rate and reconstruction accuracy, the proposed method in Algorithm 2 employs a continuous strategy where both parameters $\alpha_{1}$ and $\alpha_{2}$ are initialized to small values, and are gradually increased until the stopping criteria is satisfied.

**Figure 3:**
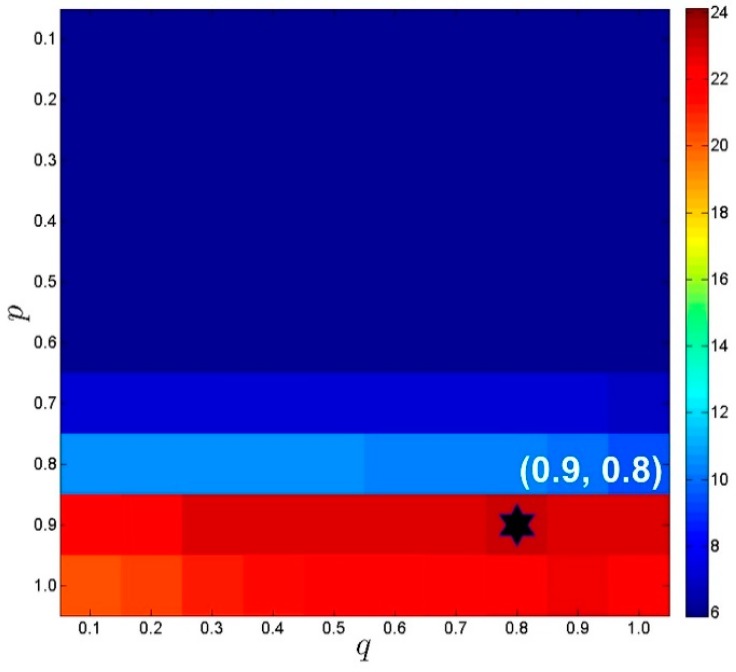
The influences of different parameters (p,q) on image reconstruction for axial cardiac dataset. The undersampled (k,t)-space measurements were generated using 12 radial projections. The optimal values of (p,q) were determined using the quality metric SER.

The parameters p and q are respectively related to the non-convex low-rank and sparsity constraints. They play an important role in the improvement of reconstructed image quality. Exhaustive experiments in this section were performed to manually determine the optimal choices of p and q. Take the in vivo axial cardiac dataset as an example, the undersampled (k,t)-space measurements were generated using 12 radial projections. A series of searches within a predefined range of parameters was performed to select the optimal values. For the sake of simplicity, the predefined range [0.1, 0.2,⋯, 1.0] was set for both p and q. As shown in Figure 3, the proposed **k**-*t* NCRPCA is more sensitive to p than q. It means that non-convex low-rank constraint proposed in method (10) can significantly affect the reconstructed image quality. According to the quantitative results, the resulting optimal parameters were set as p=0.9 and q=0.8 for axial cardiac dataset. These manually-selected parameters were also used for coronal cardiac imaging experiments. The reconstruction results under current parameter settings were consistently promising in our numerical experiments. Further study on automatically calculating the above-discussed parameters for **k**-*t* NCRPCA is our future work.

The standard for the stopping criteria in Algorithm 2 is that the relative change of F is sufficiently small, i.e.,

(27) $$\|F^{k+1}-F^{k}{\|}_{2}/\|F^{k}{\|}_{2}\leq \epsilon \;\mathrm{or}\;k>k_{\max}$$

where ϵ and $k_{\max}$, respectively, denote the predefined tolerance parameter and maximum number of iterations.

For all reconstruction experiments, $\epsilon =10^{-4}$ and $k_{\max}=300$ were respectively set in Equation (27). It is fair to compare the experimental results because the results of other competing methods are all generated using the authors’ codes with the parameters manually optimized.

### 4.3. Comparisons on In Vivo Axial Cardiac Dataset

In order to evaluate the reconstruction performance, the proposed **k**-*t* NCRPCA will be compared to other dynamic reconstruction techniques, i.e., zero-filled inverse Fourier transform (ZF-IDFT), **k**-*t* FOCUSS [31,32], **k**-*t* SLR [38] and **k**-*t* RPCA [44]. The good performance of **k**-*t* FOCUSS benefits from the sparse properties of dynamic MRI. **k**-*t* SLR took into account both sparsity and spectral priors to accelerate dynamic imaging. **k**-*t* RPCA formulated dynamic reconstruction as a least-squares optimization problem which was regularized by a low-rank plus sparse prior.

**Figure 4:**
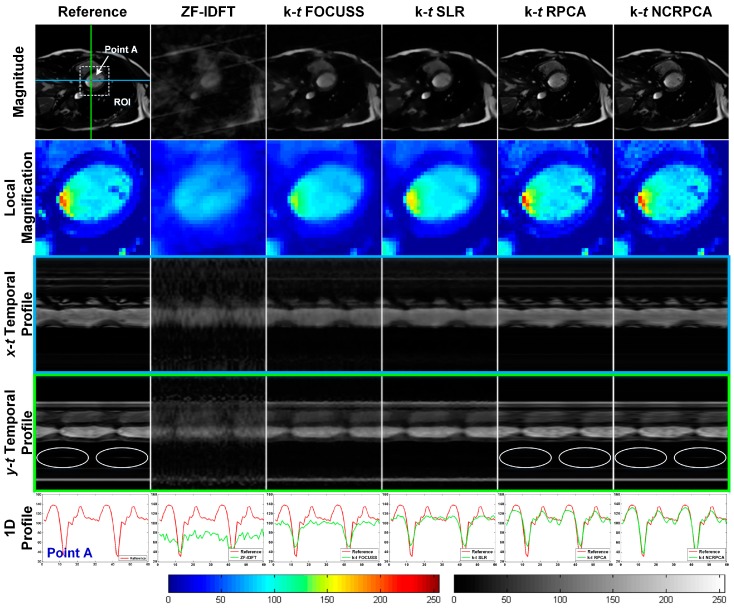
Qualitative results for in vivo axial cardiac dataset with 8 radial projections. From top to bottom: time-frame magnitude images, local magnification views (corresponding to the white square on the reference image), *x*-*t* temporal profiles, *y*-*t t*emporal profiles, and 1D profiles (according to the point A on the reference image). Left colormap refers to local magnification views. Right colormap refers to magnitude images and temporal profiles. (The images are best viewed in full-screen mode.)

Figure 4 visually illustrate the time-frame magnitude images, local magnification views, temporal profiles and 1D profiles for the in vivo axial cardiac dataset with 8 radial projections (correspond to undersampling factor of ~25). It can be observed that ZF-IDFT generates the worst perceptual results. The loss of information details severely degrades the image quality. **k**-*t* FOCUSS and **k**-*t* SLR could reconstruct the main geometrical structures but seemed to overcome some fine details. As shown by the white ellipse in *y-t t*emporal profiles, both **k**-*t* RPCA and **k**-*t* NCRPCA perform well in preserving fine image details to guarantee high image quality. For the sake of comparison, the reconstruction performance is further confirmed by the 1D profiles. It is easy to see that the intensity values of **k**-*t* NCRPCA are more structurally similar to the fully sampled reference image. In contrast, **k**-*t* FOCUSS, **k**-*t* SLR and **k**-*t* RPCA lead to accuracy loss in determining the temporal profiles due to reconstruction biases. The good performance of **k**-*t* NCRPCA over other methods can possibly be attributed to the fact that non-convex minimization benefits for improving image reconstruction from fewer measurements. 

**Figure 5:**
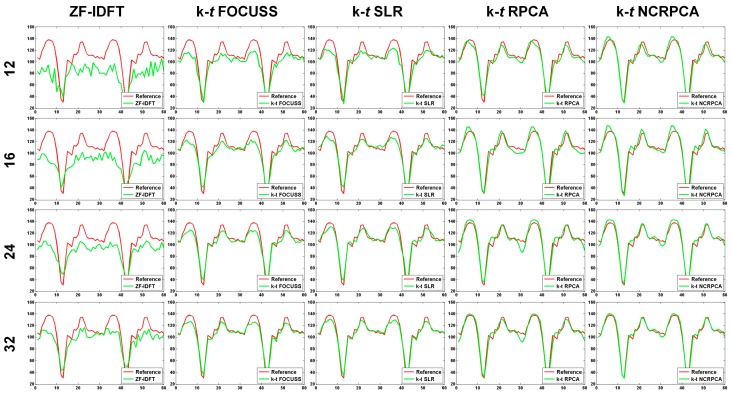
1D profiles respectively generated by ZF-IDFT, **k**-*t* FOCUSS, **k**-*t* SLR, **k**-*t* RPCA and **k**-*t* NCRPCA for different number of radial projections (i.e., 12, 16, 24 and 32). (The images are best viewed in full-screen mode.)

To further evaluate the reconstruction result, more 1D profiles for different number of radial projections are visually illustrated in Figure 5. Similar to our findings in Figure 4, we can find significant performance improvement of **k**-*t* NCRPCA in comparison to other competing methods. The quantitative results are given in Table 1. It can be concluded that our proposed **k**-*t* NCRPCA achieves the best reconstruction results in all cases.

### 4.4. Comparisons on In Vivo Coronal Cardiac Dataset

**Figure 6:**
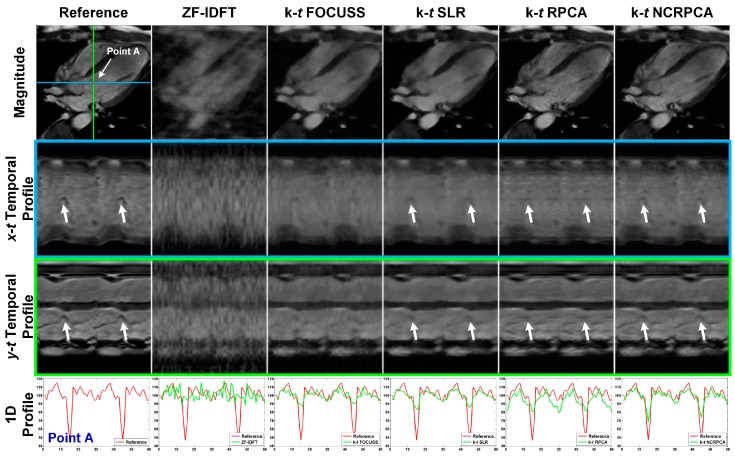
Qualitative results for in vivo coronal cardiac dataset with eight radial projections. From top to bottom: time-frame magnitude images, *x*-*t* temporal profiles, *y*-*t t*emporal profiles, and 1D profiles (according to the point A on the reference image). (The images are best viewed in full-screen mode.)

In Figure 6, we compared our **k**-*t* NCRPCA with ZF-IDFT, **k**-*t* FOCUSS, **k**-*t* SLR and **k**-*t* RPCA on an in vivo coronal cardiac dataset for 8 radial projections. It is impossible to satisfactorily reconstruct the main geometical structures using ZF-IDFT. As shown by the arrows in *x-t* and *y-t* temporal profiles, **k**-*t* FOCUSS, **k**-*t* SLR and **k**-*t* RPCA failed to achieve high quality temporal profiles due to over smoothing. The loss of useful visual information could lead to visual quality degradation. In contrast, **k**-*t* NCRPCA performs well in feature-preserving image reconstruction even for highly undersampled (k,t)-space measurements.

**Figure 7:**
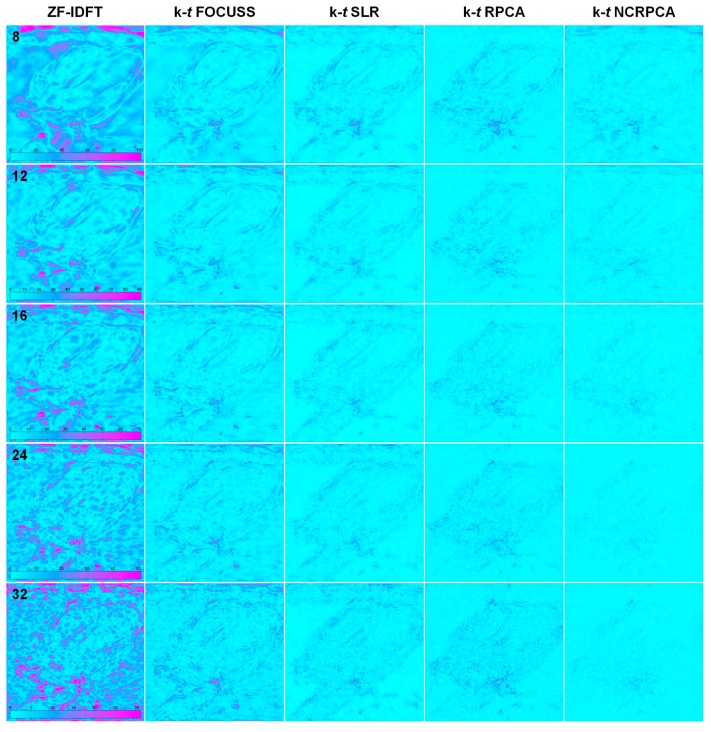
Visual appearance of error images respectively generated by ZF-IDFT, **k**-*t* FOCUSS, **k**-*t* SLR, **k**-*t* RPCA and **k**-*t* NCRPCA for different number of radial projections (i.e., 8, 12, 16, 24 and 32). (The images are best viewed in full-screen mode.)

For 1D profiles, **k**-*t* NCRPCA is able to preserve more structural information. For the sake of visual comparison, we generated the error images resulting from the absolute differences between the fully sampled reference and reconstructed images shown in Figure 7. To achieve high quality reconstruction, the error images should include as little geometrical information as possible. As can be observed, the geometrical structures are noticeable in error images generated by ZF-IDFT. **k**-*t* FOCUSS, **k**-*t* SLR and **k**-*t* RPCA tend to oversmooth some image details. In contrast, our **k**-*t* NCRPCA generates error images with more random and smaller absolute differences, which result in benefit to image quality improvement. The advantage of **k**-*t* NCRPCA is further confirmed by the SER comparison in Table 2. 

The main benefit of our proposed method is that it takes full advantage of non-convex low-rank and sparsity constraints. Current studies have illustrated that non-convex minimization could provide consistently better performance over convex minimization [32,39,40]. Thus, our method is able to effectively preserve fine details while suppressing undesirable artifacts for dynamic MRI reconstruction from undersampled (k,t)-space measurements.

### 4.5. Algorithm Convergence and Robustness

**Figure 8:**
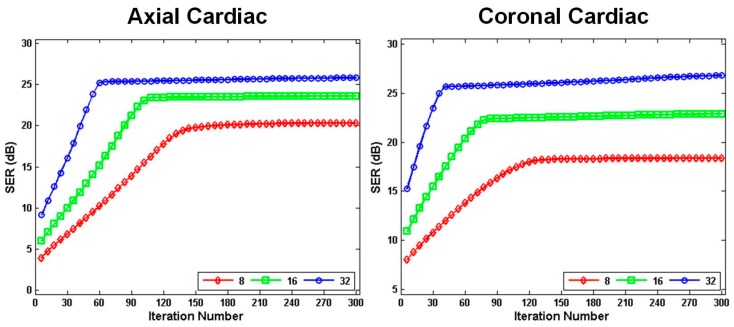
Convergence property of the proposed **k**-*t* NCRPCA. Progression of the objective quality metric SER for in vivo axial (left) and coronal (right) cardiac datasets with respect to iteration number. The undersampled (k,t)-space measurements were generated from 8, 16 and 32 radial projections.

The convergence of the proposed **k**-*t* NCRPCA was investigated on in vivo axial and coronal cardiac datasets. The undersampled (k,t)-space measurements for these two datasets were generated from 8, 16 and 32 radial projections. It is difficult to establish the theoretical convergence result because of the non-convex and non-smooth objective Function (10). For the sake of simplicity, the convergence property of the proposed algorithm was analyzed empirically. As shown in Figure 8, the objective quality metric SER is visually displayed as a function of iteration number. It is observed that with the increase of iteration number, SER values increase quickly at the first few iterations and then become stable. These observations illustrate that the convergence property of the proposed method, summarized in Algorithm 2, can be guaranteed for undersampled dynamic MRI reconstruction. Furthermore, it is obvious that the proposed method is convergent even for robust reconstruction from highly undersampled (k,t)-space measurements (i.e., 8 radial projection in experiments). The experiments on in vivo axial and coronal cardiac datasets fully illustrate the robustness of the proposed method.

## 5. Conclusions

In this study, a new constrained imaging method (termed **k**-*t* NCRPCA) was proposed to accelerate dynamic MRI. It effectively integrated both non-convex low-rank and sparsity constraints into a unified mathematical framework. The resulting non-convex and non-smooth optimization problem was effectively solved using an ADMM-based optimization algorithm. Numerous experiments have been conducted on two in vivo cardiac datasets to compare **k**-*t* NCRPCA with other state-of-the-art reconstruction methods. The experimental results have demonstrated the superior performance of **k**-*t* NCRPCA in terms of both quantitative and qualitative image quality evaluations. Therefore, there is a strong incentive to extend **k**-*t* NCRPCA to effectively accelerate dynamic MRI in clinical practice.

## Figures and Tables

**Table 1:** SSIM/SER comparison of various reconstruction methods on in vivo axial cardiac MRI dataset for different number of radial projections.

Projections	ZF-IDFT	k-*t* FOCUSS	k-*t* SLR	k-*t* RPCA	k-*t* NCRPCA
8	0.2555/3.4603	0.7376/11.2002	0.8802/12.6634	0.9476/19.6096	0.9503/20.1538
12	0.3088/4.4626	0.7969/12.9951	0.9257/15.6441	0.9760/22.0393	0.9784/22.9995
16	0.3608/5.3026	0.8518/15.2740	0.9518/17.7428	0.9769/22.9016	0.9789/23.7973
24	0.4408/6.7989	0.9004/17.0383	0.9671/20.3897	0.9828/24.0802	0.9942/24.9276
32	0.4981/8.1953	0.9257/20.4791	0.9831/22.8358	0.9870/25.1264	0.9956/25.8280

**Table 2:** SSIM/SER comparison of different reconstruction methods on in vivo coronal cardiac MRI dataset for different number of radial projections.

Projections	ZF-IDFT	k-*t* FOCUSS	k-*t* SLR	k-*t* RPCA	k-*t* NCRPCA
8	0.3281/7.3112	0.7041/16.1893	0.8328/16.9640	0.7948/17.5719	0.8268/17.8084
12	0.4306/8.7548	0.8099/17.9173	0.8894/18.8529	0.8682/20.2187	0.8937/20.6879
16	0.4974/9.9446	0.8618/19.6515	0.9300/20.8879	0.9134/21.9271	0.9384/22.6999
24	0.5929/11.9903	0.9064/21.9681	0.9546/23.1806	0.9383/23.5353	0.9768/25.1972
32	0.6753/13.9492	0.9489/24.2931	0.9757/25.7613	0.9674/25.4710	0.9887/27.2034

## References

[1] Edelman R.R., Warach S. (1993). Magnetic resonance imaging. N. Engl. J. Med..

[2] Xiong N., Vasilakos A.V., Yang L.T., Song L., Pan Y., Kannan R., Li Y. (2009). Comparative analysis of quality of service and memory usage for adaptive failure detectors in healthcare systems. IEEE J. Sel. Areas Commun..

[3] O'connor J.P., Jackson A., Parker G.J., Roberts C., Jayson G.C. (2012). Dynamic contrast-enhanced MRI in clinical trials of antivascular therapies. Nat. Rev. Clin. Oncol..

[4] Xiong N., Jia X., Yang L.T., Vasilakos A.V., Li Y., Pan Y. (2010). A distributed efficient flow control scheme for multirate multicast networks. IEEE Trans. Parallel Distrib. Syst..

[5] Harada T., Tsuji Y., Mikami Y., Hatta Y., Sakamoto A., Ikeda T., Kubo T. (2010). The clinical usefulness of preoperative dynamic MRI to select decompression levels for cervical spondylotic myelopathy. Magn. Reson. Imaging.

[6] Leach M.O., Morgan B., Tofts P.S., Buckley D.L., Huang W., Horsfield M.A., Whitcher B. (2012). Imaging vascular function for early stage clinical trials using dynamic contrast-enhanced magnetic resonance imaging. Eur. Radiol..

[7] Xia Z., Xiong N.N., Vasilakos A.V., Sun X. (2016). EPCBIR: An efficient and privacy-preserving content-based image retrieval scheme in cloud computing. Inf. Sci..

[8] Lingala S.G., DiBella E., Adluru G., McGann C., Jacob M. (2013). Accelerating free breathing myocardial perfusion MRI using multi coil radial **k**-*t* SLR. Phys. Med. Biol..

[9] Denney T.R., Prince J.L. (1995). Reconstruction of 3-D left ventricular motion from planar tagged cardiac MR images: An estimation theoretic approach. IEEE Trans. Med. Imaging.

[10] Thesen S., Heid O., Mueller E., Schad L.R. (2000). Prospective acquisition correction for head motion with image-based tracking for real-time fMRI. Magn. Reson. Med..

[11] Mahapatra D., Buhmann J.M. (2014). Prostate MRI segmentation using learned semantic knowledge and graph cuts. IEEE Trans. Biomed. Eng..

[12] Xia Z., Wang X., Sun X., Liu Q., Xiong N. (2016). Steganalysis of LSB matching using differences between nonadjacent pixels. Multimed. Tools Appl..

[13] Chen Y., Zhang H., Zheng Y., Jeon B., Wu Q.J. (2016). An improved anisotropic hierarchical fuzzy c-means method based on multivariate student t-distribution for brain MRI segmentation. Pattern Recognit..

[14] Zhang Y., Dong Z., Wu L., Wang S. (2011). A hybrid method for MRI brain image classification. Expert Syst. Appl..

[15] Wen X., Shao L., Xue Y., Fang W. (2015). A rapid learning algorithm for vehicle classification. Inf. Sci..

[16] Li Z., Lai Z., Xu Y., Yang J., Zhang D. (2017). A locality-constrained and label embedding dictionary learning algorithm for image classification. IEEE Trans. Neural Netw. Learn. Syst..

[17] Liu R.W., Shi L., Yu S.C., Wang D. (2015). A two-step optimization approach for nonlocal total variation-based Rician noise reduction in magnetic resonance images. Med. Phys..

[18] Baselice F., Ferraioli G., Pascazio V., Sorriso A. (2017). Bayesian MRI denoising in complex domain. Magn. Reson. Imaging.

[19] Pizzolato M., Boutelier T., Deriche R. (2017). Perfusion deconvolution in DSC-MRI with dispersion-compliant bases. Med. Image Anal..

[20] Koh T.S., Cheong D.L.H., Hou Z. (2011). Issues of discontinuity in the impulse residue function for deconvolution analysis of dynamic contrast-enhanced MRI data. Magn. Reson. Med..

[21] Han S., Paulsen J.L., Zhu G., Song Y., Chun S., Cho G., Ackerstaff E., Koutcher J.A., Cho H. (2012). Temporal/spatial resolution improvement of in vivo DCE-MRI with compressed sensing-optimized FLASH. Magn. Reson. Imaging.

[22] Pain F., Besret L., Vaufrey F., Grégoire M.C., Pinot L., Gervais P., Ploux L., Bloch G., Mastrippolito R., Lanièce P. (2002). In vivo quantification of localized neuronal activation and inhibition in the rat brain using a dedicated high temporal-resolution β+-sensitive microprobe. Proc. Natl. Acad. Sci. USA.

[23] Boschetto D., Di Prima P., Castellaro M., Bertoldo A., Grisan E. Baseline constrained reconstruction of DSC-MRI tracer kinetics from sparse fourier data. Proceedings of the IEEE International Symposium on Biomedical Imaging.

[24] Candes E.J., Romberg J., Tao T. (2006). Robust uncertainty principles: Exact signal reconstruction from highly incomplete frequency information. IEEE Trans. Inf. Theory.

[25] Donoho D.L. (2006). Compressed sensing. IEEE Trans. Inf. Theory.

[26] Lustig M., Donoho D., Pauly J.M. (2007). Sparse MRI: The application of compressed sensing for rapid MR imaging. Magn. Reson. Med..

[27] Lustig M., Donoho D.L., Santos J.M., Pauly J.M. (2008). Compressed sensing MRI. IEEE Signal Process. Mag..

[28] Zhang Y., Peterson B.S., Ji G., Dong Z. (2014). Energy preserved sampling for compressed sensing MRI. Comput. Math. Method Med..

[29] Zhang Y., Dong Z., Phillips P., Wang S., Ji G., Yang J. (2015). Exponential wavelet iterative shrinkage thresholding algorithm for compressed sensing magnetic resonance imaging. Inf. Sci..

[30] Cai N., Wang S., Zhu S., Liang D. (2013). Accelerating dynamic cardiac MR imaging using structured sparse representation. Comput. Math. Methods Med..

[31] Jung H., Sung K., Nayak K.S., Kim E.Y., Ye J.C. (2009). k-t FOCUSS: A general compressed sensing framework for high resolution dynamic MRI. Magn. Reson. Med..

[32] Jung H., Ye J.C., Kim E.Y. (2007). Improved k-t BLAST and k-t SENSE using FOCUSS. Phys. Med. Biol..

[33] Feng L., Srichai M.B., Lim R.P., Harrison A., King W., Adluru G., Dibella E.V., Sodickson D.K., Otazo R., Kim D. (2013). Highly accelerated real-time cardiac cine MRI using k-t SPARSE-SENSE. Magn. Reson. Med..

[34] Candes E.J., Recht B. (2009). Exact matrix completion via convex optimization. Found. Comput. Math..

[35] Otazo R., Candes E., Sodickson D.K. (2015). Low-rank plus sparse matrix decomposition for accelerated dynamic MRI with separation of background and dynamic components. Magn. Reson. Med..

[36] Liu Q., Lai Z., Zhou Z., Kuang F., Jin Z. (2016). A truncated nuclear norm regularization method based on weighted residual error for matrix completion. IEEE Trans. Image Process..

[37] Fang X., Xu Y., Li X., Lai Z., Wong W.K. (2016). Robust semi-supervised subspace clustering via non-negative low-rank representation. IEEE Trans. Cybern..

[38] Lingala S.G., Hu Y., DiBella E., Jacob M. (2011). Accelerated dynamic MRI exploiting sparsity and low-rank structure: k-t SLR. IEEE Trans. Med. Imaging.

[39] Zhao B., Haldar J.P., Christodoulou A.G., Liang Z.P. (2012). Image reconstruction from highly undersampled (k,t)-space data with joint partial separability and sparsity constraints. IEEE Trans. Med. Imaging.

[40] Candes E.J., Li X.D., Ma Y., Wright J. (2011). Robust principal component analysis?. J. ACM.

[41] Yuan X., Yang J. (2013). Sparse and low-rank matrix decomposition via alternating direction methods. Pac. J. Optim..

[42] Tao M., Yuan X.M. (2011). Recovering low-rank and sparse components of matrices from incomplete and noisy observations. SIAM J. Optim..

[43] Wright J., Ganesh A., Min K.R., Ma Y. (2013). Compressive principal component pursuit. Inf. Inference.

[44] Trémoulhéac B., Dikaios N., Atkinson D., Arridge S.R. (2014). Dynamic MR image reconstruction-separation from undersampled (k,t)-space via low-rank plus sparse prior. IEEE Trans. Med. Imaging.

[45] Majumdar A., Ward R.K. (2011). An algorithm for sparse MRI reconstruction by Schatten p-norm minimization. Magn. Reson. Imaging.

[46] Trzasko J., Manduca A. (2009). Highly undersampled magnetic resonance image reconstruction via homotopic l0-minimization. IEEE Trans. Med. Imaging.

[47] Majumdar A. (2013). Improved dynamic MRI reconstruction by exploiting sparsity and rank-deficiency. Magn. Reson. Imaging.

[48] Zhang Y., Sun X., Wang B. (2016). Efficient algorithm for k-barrier coverage based on integer linear programming. China Commun..

[49] Mehranian A., Reader A. (2016). Non-convex joint-sparsity regularization for synergistic PET and SENSE MRI reconstruction. J. Nucl. Med..

[50] Ding Y., Selesnick I.W. (2015). Artifact-free wavelet denoising: Non-convex sparse regularization, convex optimization. IEEE Signal Process. Lett..

[51] Zhang J., Xiong R., Zhao C., Zhang Y., Ma S., Gao W. (2016). CONCOLOR: Constrained non-convex low-rank model for image deblocking. IEEE Trans. Image Process..

[52] Chartrand R. (2007). Exact reconstruction of sparse signals via nonconvex minimization. IEEE Signal Process. Lett..

[53] Chartrand R., Staneva V. (2008). Restricted isometry properties and nonconvex compressive sensing. Inverse. Probl..

[54] Yuan C., Sun X., Lv R. (2016). Fingerprint liveness detection based on multi-scale LPQ and PCA. China Commun..

[55] Gasso G., Rakotomamonjy A., Canu S. (2009). Recovering sparse signals with a certain family of nonconvex penalties and DC programming. IEEE Trans. Signal Process..

[56] Liu R.W., Shi L., Huang W., Xu J., Yu S.C.H., Wang D. (2014). Generalized total variation-based MRI Rician denoising model with spatially adaptive regularization parameters. Magn. Reson. Imaging.

[57] Lai Z., Xu Y., Chen Q., Yang J., Zhang D. (2014). Multilinear sparse principal component analysis. IEEE Trans. Neural Netw. Learn. Syst..

[58] Zhou X., Yang C., Zhao H., Yu W. (2015). Low-rank modeling and its applications in image analysis. ACM Comput. Surv..

[59] Boyd S., Parikh N., Chu E., Peleato B., Eckstein J. (2011). Distributed optimization and statistical learning via the alternating direction method of multipliers. Found. Trends Mach. Learn..

[60] Cai J.F., Candes E.J., Shen Z.W. (2010). A singular value thresholding algorithm for matrix completion. SIAM J. Optim..

[61] Lu Y., Lai Z., Xu Y., Li X., Zhang D., Yuan C. (2016). Low-rank preserving projections. IEEE Trans. Cybern..

[62] Donoho D.L. (1995). De-noising by soft-thresholding. IEEE Trans. Inf. Theory.

[63] Daubechies I., Defrise M., De Mol C. (2004). An iterative thresholding algorithm for linear inverse problems with a sparsity constraint. Commun. Pure Appl. Math..

[64] Zuo W.M., Meng D.Y., Zhang L., Feng X.C., Zhang D. A generalized iterated shrinkage algorithm for non-convex sparse coding. Proceedings of the IEEE International Conference on Computer Vision.

